# Supplementary material for: Inequality in the Utilization of Breast Cancer Screening between Women with and without Disabilities in Taiwan: A Propensity-Score-Matched Nationwide Cohort Study
Source: Int J Environ Res Public Health. 2022 Apr 26;19(9):5280. doi: 10.3390/ijerph19095280 (PMC9104314; doi:10.3390/ijerph19095280)
Supplement: Supplementary file 1 [file ijerph-19-05280-s001.zip › ijerph-1656457-supplementary.pdf]

Supplementary Table S1. Characteristics of women with disabilities before matching.

Variables	N	%
**Total**	125,148	100.00
**Disability type**		
Moving functional limitation	55,572	44.41
Visual impairment	8,240	6.58
Hearing impairment	12,000	9.59
Speech disorder	922	0.74
Intellectual disability	4,399	3.52
Multiple disabilities	9,619	7.69
Dysfunction of primary organs	19,048	15.22
Facial impairment	214	0.17
Dementia	1,657	1.32
Congenital disorders	24	0.02
Chronic mental health conditions	12,920	10.32
Balance disorder	322	0.26
Intractable epilepsy	151	0.12
Rare diseases	42	0.03
Other [a]	18	0.01
**Severity of disability**		
Mild	45,436	36.31
Moderate	39,498	31.56
Severe	20,274	16.20
Very severe	19,940	15.93
**Age**		
50-59 years	69,570	55.59
60-69 years	55,578	44.41
**Monthly salary (NT$)**		
Low-income households	4,475	3.58
≦17,280	7,297	5.83
17,281-22,800	54,715	43.72
22,801-28,800	25,295	20.21
28,801-36,300	16,742	13.38
≧36,301	16,624	13.28
**Urbanization level**		
1	25,550	20.42
2	37,658	30.09
3	18,781	15.01
4	22,928	18.32
5	4,330	3.46

6	8,465	6.76
7	7,436	5.94
**CCI**		
0	53,251	42.55
1	26,739	21.37
2	19,252	15.38
≧3	25,906	20.70
**Adults' preventive care service**		
No	88,377	70.62
Yes	36,771	29.38

[a] Including autism, chromosomal abnormalities, congenital metabolic abnormalitie.
